# Enhanced Monitoring of the Preterm Infant during Stabilization in the Delivery Room

**DOI:** 10.3389/fped.2016.00030

**Published:** 2016-03-31

**Authors:** Daragh Finn, Geraldine B. Boylan, C. Anthony Ryan, Eugene M. Dempsey

**Affiliations:** ^1^Department of Paediatrics and Child Health, University College Cork, Cork, Ireland; ^2^Irish Centre for Fetal and Neonatal Translational Research, University College Cork, Cork, Ireland

**Keywords:** preterm, newly born infant, monitoring, neuromonitoring, delivery-room, stabilization, resuscitation

## Abstract

Monitoring of preterm infants in the delivery room (DR) remains limited. Current guidelines suggest that pulse oximetry should be available for all preterm infant deliveries, and that if intubated a colorimetric carbon dioxide detector should provide verification of correct endotracheal tube placement. These two methods of assessment represent the extent of objective monitoring of the newborn commonly performed in the DR. Monitoring non-invasive ventilation effectiveness (either by capnography or respiratory function monitoring) and cerebral oxygenation (near-infrared spectroscopy) is becoming more common within research settings. In this article, we will review the different modalities available for cardiorespiratory and neuromonitoring in the DR and assess the current evidence base on their feasibility, strengths, and limitations during preterm stabilization.

## Key Points

Current ILCOR Guidelines recommend for all preterm infant deliveries:

- Pulse oximetry for SpO_2_ monitoring and titration of O_2_ therapy
- Pulse oximetry and consideration of ECG as an adjunct for heart rate monitoring
- CO_2_ detectors to verify correct endotracheal tube positioning

## Introduction

In recent decades, we have witnessed a significant increase in the number of monitoring options for preterm infants in the neonatal intensive care unit (NICU) setting. Examples include cardiac (echocardiography and non-invasive cardiac output monitoring), respiratory (capnography and respiratory function monitoring), and neurological monitoring [electroencephalography (EEG) and near-infrared spectroscopy (NIRS)]. By contrast, over the same timeframe, monitoring of preterm infants in the delivery room (DR) has changed very little, other than the introduction of pulse oximetry approximately 10 years ago.

As adjuncts to clinical monitoring during initial preterm stabilization in the DR, the recent 2015 ILCOR recommendations advise the use of two objective assessment tools: (1) pulse oximetry (with or without ECG) to regulate oxygen delivery and (2) exhaled carbon dioxide (CO_2_) detectors for confirmation of correct endotracheal (ET) tube placement (1). These two devices generate real-time accurate physiological data and, if recorded, chronicle changing observations over time. The information provided assists in clinical decision-making and has the potential to improve both short- and long-term outcomes for preterm infants.

The relative lack of monitoring options in the DR is both a reflection of the difficulties in acquiring the information and interpreting these data for decision-making in real-time. As Bradley and Field reflected, “not all that is measurable is of value, and not all that is of value can be measured” (2). Introducing new monitoring devices into clinical care remains challenging. Feasibility studies are initially required to assess whether new devices can be applied safely, useful information acquired, interpreted, and acted upon in the simulation environment. Human factors need to be considered and evaluated prior to introduction of new technology into the clinical environment. This may involve additional team training, possibly in the form of newborn resuscitation simulations, and it should be highlighted that new devices should not distract team members from complying with current resuscitation recommendations. Preterm adaptation represents a unique physiological time in life. Ideally, both short- and long-term benefits should be evaluated by randomized controlled trials prior to the introduction of a new device into routine clinical care.

This review will present an overview of current methods of preterm newborn monitoring in the DR, from simple clinical evaluation to the potential role of newer monitoring devices, including monitoring cerebral activity and cerebral oxygenation during the first minutes of life (Table 1).

**Table 1:** **Summary of monitoring devices**.

Variable	Monitor	Data acquisition feasible	Normative values established^a^	Comments	Strength of recommendation
SpO_2_	Pulse oximeter	+	+	Accurate but unable to detect hyperoxemia	Class I
HR	Pulse oximeter	+	+	Accurate but time delay in data acquisition	Class I
	ECG	+	+	Rapid accurate data acquisition	Class I
Peripheral perfusion	Echocardiography/NICOM	+	−	Not assessed in preterm infants	Class III
	Perfusion index	+	+	Normative values highly variable in newborns	Class III
ETT position	CO_2_ detector	+	n/a	Reduces time to confirmation of correct placement	Class I
Facemask ventilation effectiveness	CO_2_ detector	+	n/a	Reduces mask leak and obstruction	Class IIa
				Further RCTs required	
	Capnography	+	−	Reduces mask leak and obstruction	Class IIa
				Further RCTs required	
	Respiratory function monitor	+	−	Reduces mask leak and obstruction	Class IIa
				Further RCTs required	
Cerebral oxygenation	NIRS	+	+	Advise as part of further RCTs	Class IIb
Cerebral activity	EEG	+	−	Advise further feasibility trials and establishment of normative reference values	Class III

*^a^For preterm infants, <32 weeks gestation in the immediate newborn period*.

## Historical Context: Clinical Assessment of the Preterm Infant and the Apgar Score

Dr. Virginia Apgar, in 1953, was the first to describe newborn monitoring in the DR in a methodical manner. *The Apgar score* is the sum of values based on the newborn respiratory (respirations, skin color), cardiovascular [heart rate (HR), skin color], and neurological (muscle tone, reflex irritability) systems (3). With the exception of HR, all of the variables are based on visual inspection of the infant and as such are somewhat subjective. Large cohort studies identified that 5-min Apgar scores of <7 were associated with increased risk of neonatal death and cerebral palsy in both term and preterm infants (4–6), indicating that early clinical assessments may be reliable and meaningful for newborn infants. On addressing the interrater variability of the score, Apgar reported that, “When two or more people decide independently, we find a range of one value above or below a decided score to be the widest variation” (7, 8). Currently, Apgar scores remain central to our interpretation of a newborn’s condition at birth. They are routinely assigned to all infants in the immediate postnatal period and are usually collected as part of research trials both to assess baseline characteristics of study participants and in some cases as outcome measures. Newborn resuscitation guidelines advise initiating support during infants’ transition based on the assessment of respirations, tone, and HR, which are all components of the Apgar score (9–11).

However, more recent studies have shown poor inter- and intrarater reliability with regard to Apgar score assignment, especially when the infant is preterm or ventilated (12, 13). The ability of the 5-min Apgar score to predict outcome seem less likely than previously thought. Singh et al. have shown that in very preterm infant delivery, there is no Apgar score cutoff below which “a burdensome outcome was assured or above which an unscathed outcome was likely.” Five-minute Apgar score and HR values also displayed poor sensitivity and specificity for either survival or survival without disability (14). Manley and colleagues asked clinicians to predict the outcome of preterm infants (<26 weeks gestation) based on their clinical appearance in the DR, at prespecified time points of 20 s, 2 min, and 5 min. This study was based on video recordings of the preterm infants, and monitors displaying HR and oxygen saturation (SpO_2_) values were visible. Trainees and staff neonatologists predicted infant survival poorly at each time point. The authors concluded that neonatologists’ “reliance on initial appearance and early response to resuscitation in predicting survival for extremely premature infants is misplaced” (15).

Updated Apgar scoring systems have been proposed and allow for more appropriate descriptions of the condition of the preterm infant at birth. The *Combined-Apgar score* reports the infants’ score in each of the five components of the Apgar score (specified Apgar score), and the interventions required to achieve this score (expanded Apgar score) (16, 17). This combined-Apgar score has been shown to be superior in predicting outcome in preterm infants when compared to the conventional-Apgar score (18). However, this updated scoring system has yet to be universally adopted and the relevance of conventional Apgar scores in preterm infants remains limited.

## Oxygen Saturation Monitoring

Clinically, infants transition from blue (cyanotic) to pink (normal oxygen saturations) in color during uncomplicated newborn transition. O’Donnell and colleagues assessed clinical perceptions of newborn infant color in the DR (19). They found wide variation in observations and concluded that, “clinical assessment of a newborn infant’s color may be unreliable.” Assessment of arterial oxygen saturation by *pulse oximetry* is based on the Beer–Lambert law that relates the attenuation of light to the properties of the materials through which the light is traveling; and photoplethysmography, a non-invasive optical technique used to detect blood volume changes in the microvascular bed of the tissue (20). Aoyagi and Kishi, who realized that oxygenated hemoglobin absorbs more light at infrared wavelengths and deoxygenated hemoglobin absorbs more light at red wavelengths, developed arterial oxygen saturation monitoring by pulse oximetry in 1972. The changes during systole and diastole in the ratio of red and infrared light energy absorption are used to produce the pulse oxygen saturation (21).

The device was first commercialized in 1981, and the use of pulse oximetry for continuous oxygen monitoring in newborns was first described in 1986 (22). The clinical benefits of pulse oximetry were quickly recognized, and it has become the mainstay of non-invasive, continuous SpO_2_ monitoring in newborns (23). Oxygen saturation monitoring of preterm infants is now standard in the DR, and these values serve as a guide to stabilization (22, 23), and the titration of oxygen therapy in preterm newborn stabilization is now routine to achieve targeted saturations by 10 min of age (24, 25). Dawson and colleagues have published oxygen saturation percentile charts for the first 10 min of life (24). In their study of over 450 infants, they observed the SpO_2_ values of preterm infants increased at a slower pace than term infants. At 5 min, the median (interquartile range) SpO_2_ was 86% (80–92) in preterm and 92% (83–96) in term infants. They have published three sets of percentile charts based on gestation (>37, 32–37, and <32 weeks), which may guide neonatal teams in titrating oxygen therapy in the DR. However, these ranges were developed in a cohort of infants born when immediate cord clamping was frequently practiced following delivery. Smit and colleagues assessed over 100 term infants with delayed cord clamping and concluded that the Dawson curves are still relevant; however, they documented higher initial SpO_2_ values, lower HRs, and a slower increase over the first 3 min of life (26).

Pulse oximetry has gained widespread acceptance in neonatal care over the past three decades because of its reliability, ease of use, and lack of heat-related complications. The main physiological limitation of pulse oximetry is the inability to detect hyperoxemia in the higher SpO_2_ range (>90%) because of the shape of the oxygen–hemoglobin dissociation curve. Thus, relatively small increases in SpO_2_ can be associated with a large increase in PaO_2_ (27–29). This is particularly important for preterm infants receiving supplemental oxygen because of their vulnerability to oxygen toxicity and oxidative stress (30). Despite this limitation, pulse oximetry is the gold standard for monitoring oxygen saturation during preterm infant stabilization, and it should be used following all preterm deliveries.

## Heart Rate Monitoring

Monitoring HR helps to guide newborn transition and the need for intervention in the DR. Current recommendations advise that HR should be assessed clinically, and if positive pressure ventilation is commenced HR should be monitored by pulse oximetry, with the option of additional ECG monitoring (1).

Although *clinical assessment of HR* by auscultation at the apex is more accurate than assessment by palpation of the umbilicus (31), all clinical assessments may misrepresent the actual HR. Kamlin et al. compared palpation and auscultation of HR to ECG determined HR in term newborns in the DR. They found that clinical assessments were inaccurate, and infant HR was underestimated when compared with ECG HRs (32). Hawkes et al. studied health-care professionals as they palpated a simulated pulsating umbilicus, listened to a tapping HR, or auscultated a simulated HR. They found that while study participants performed well at identifying HR > 100 beats per minute (bpm), almost two-thirds of participants failed to recognize a HR <60 bpm for all methods of assessment (33). These findings emphasize the importance of early accurate objective HR monitoring during preterm infant transition for identification of infants who may require support or active resuscitation (HR <60 bpm).

*Pulse oximetry* provides real-time accurate information about the HR of the preterm infant (34). However, pulse oximetry values are not available immediately as the sensor takes time to apply correctly and once applied, there is a delay before the monitor provides a reading. Limb perfusion will affect the time taken to achieve a pulse oximeter HR (34). Studies that have assessed the feasibility of obtaining prompt and reliable pulse oximetry readings have reported times to signal acquisition of between 1 and 2 min after delivery (25, 35). There is conflicting evidence as to whether quicker signal acquisitions are obtained by applying the sensor to the oximeter first or applying it to the infant first. Observational studies reported that the quickest method involved turning on the pulse oximeter prior to delivery, applying the sensor to the infant’s right hand and then connecting the cable of the sensor to the oximeter. This results in mean readings within 25 s of reaching the resuscitation table in a research setting (35, 36). A recent RCT in the DR contradicted these findings and found significantly faster signal acquisition times in infants who had the sensor attached to the oximeter first (37). A limitation of pulse oximetry HR monitoring is that HRs <100 bpm are not consistently detected, and in a study by Kamlin et al. were only reported 89% of the time (34).

*ECG monitoring* can provide accurate HR values sooner than pulse oximetry following delivery (34, 38). The electrodes can be applied quickly, and there is little lapse in time waiting for monitor readings to appear. Katheria and colleagues reported that median times to acquire a signal from ECG and pulse oximetry were 4 and 32 s, respectively (38). A limitation of ECG monitoring is the risk of pulseless electrical activity being misinterpreted as HR on ECG (39). *Doppler ultrasound* blood flow HR assessments in the DR are accurate compared with clinical and pulse oximetry assessments (40). Measurements can be taken through a polyethylene bag. However, clinical experience is required for accurate assessments and continuous measurements are not practical.

ECG monitoring cannot replace the need for pulse oximetry, which is necessary for SpO_2_ monitoring. However, given that ECG monitoring is more accurate than clinical estimations, ECG may prevent unnecessary interventions secondary to falsely low clinical HR estimations. Alternatively, it could increase interventions, which may or may not be appropriate, because of earlier accurate bradycardia detection. Although awaiting further evidence, there are a few important points to be made: initiation of ECG monitoring in the DR is easily achievable, is more accurate than clinical assessment and provides HR values more expediently than pulse oximetry. Therefore, at present, our practice is to have ECG monitoring available, as an adjunct to SpO_2_ monitoring, for all preterm deliveries <32 weeks gestation and in situations where advanced resuscitation is anticipated. Clinical trials are required to assess whether ECG monitoring affects the frequency of stabilization interventions, and ultimately whether its use affects stabilization outcomes.

## Peripheral Perfusion Monitoring

Peripheral perfusion is determined by cardiac output and the caliber of the vessels transporting blood to the peripheries. Current clinical methodologies for non-invasive monitoring of peripheral perfusion include assessments of capillary refill time, peripheral temperatures, and palpation of peripheral pulses. Each method relies on subjective assessments and continuous measurements are impractical. *Blood Pressure monitoring* by Doppler and oscillometric methods are feasible in the DR and measurements for term infants have been reported (41). However, non-invasive measurements are not reliably consistent in preterm neonates, and invasive BP monitoring is not practical within the DR setting.

A recent review by Baik et al. identified four studies of *echocardiographic monitoring* during newborn stabilization (42). Each study assessed term infants only (43–46). Increased left ventricular output and stroke volume increased over the first 15 min of life (44–46) and one study reported an increase in left-to-right shunting across the ductus (46). The studies did not assess echocardiographic measurements of HR. The authors of the review concluded that echocardiographic monitoring in the DR would enhance our knowledge about “cardiac function changes” (42). However, it does not add useful clinical information during newborn stabilization, has not been assessed in preterm infants in the DR, and routine monitoring is not advised.

*Non-invasive continuous cardiac output monitoring (NICOM)* is an alternative method for assessing cardiac output in neonates. This technology is based on the assumption that changes in the resistance to electrical currents captured by electrodes on the thorax are directly related to changes in aortic volume during different stages of the cardiac cycle (30). NICOM measurements are feasible in neonates and correlate well with timed echocardiographic measurements (47, 48). However, NICOM may underestimate the actual CO value (47). Katheria and colleagues performed NICOM on 20 term infants during delayed cord clamping in the DR. They found that for the majority of infants, CO and stroke volume continued to increase in value from the second minute of life until the cord was clamped at 5 min (49). However, NICOM has yet to be assessed in preterm infants in the DR.

*Perfusion index (PI) monitoring* is a non-invasive method of assessing real-time peripheral perfusion, derived from, and displayed by the pulse oximeter. Pulse oximetry values are derived from red (660 nm) and infrared wavelengths (910–940 nm) (50). By using a third wavelength (800 nm), the overall hemoglobin content can be calculated and the pulsatile component of arterial blood can be distinguished from the non-pulsatile component (51). PI has been utilized to monitor preterm infants in a number of clinical areas (52). These include screening for congenital cardiac disease (53, 54), predicting low systemic blood flow (55), and assessing perfusion following blood transfusion (56). However, while PI values are easily obtained in the DR, and normative values for preterm infants in the first day of life have been published (57, 58), they are highly variable in the immediate newborn period, for both term and preterm infants (59). There are neither trials comparing PI and clinical assessments of peripheral perfusion in preterm care nor trials assessing whether PI monitoring affects preterm outcomes. Therefore, evidence in favor or against PI monitoring in the DR is lacking.

## Respiratory Support Monitoring

Lung aeration is a critical point in newborn transition from fetal life. Newborn preterm infants are at an increased risk of needing respiratory support following delivery. Inadequate ventilation may result in hypoxia and resultant bradycardia. International guidelines advise a stepwise approach to achieving optimal ventilation following preterm delivery and prior to escalating cardiovascular support; therefore, positive pressure ventilation is the cornerstone of neonatal resuscitation (60, 61). It is provided either by mask ventilation, single or double nasal prongs, nasopharyngeal tube, or *via* an ET tube. Adequate airway ventilation is assessed clinically by chest rise, an increase in HR, and auscultation for air entry on both sides of the lung fields during DR stabilization. Visual assessments of chest rise are not reliable (62). After initiating mask ventilation and if the clinical response is suboptimal, guidelines advise repositioning of the mask to reduce leak, and airway opening manoeuvers to combat airway obstruction. If there is no clinical improvement after such interventions a definitive airway, in the form of ET intubation is advised (1). The mnemonic MRSOPA identifies these methods, improve *M*ask seal, *R*e-position the airway, *S*uction and/or *O*pen the mouth, increase the inflation *P*ressure, and consider an *A*lternative airway.

Monitoring ventilatory efficacy in infants in the NICU is achieved by monitoring carbon dioxide (CO_2_) levels, which can be achieved by measuring either transcutaneous or exhaled CO_2_ levels. There is very little information on CO_2_ assessment at birth. Studies in the DR have focused on exhaled CO_2_ detection, either by qualitative or semiquantitative disposable *colorimetric CO_*2*_*detectors** that change color upon contact with CO_2_, or *quantitative capnography that provides a breath-by-breath end tidal CO_*2*_*measurement** (63). Quantitative capnography is achieved either by mainstream capnography that utilizes an infrared absorption technique or by side stream capnography that continuously transports a sample of gas to a sampling cell within a monitor. Both capnography methods provide a continuous visual display of CO_2_ values (capnometry) (63). Absolute CO_2_ values may be unreliable in the immediate newborn period prior to the establishment of functional residual capacity. The lungs are partially fluid filled with resultant low pulmonary blood flow, and mask leak or rebreathing may also affect absolute values. Therefore, during DR mask ventilation, members of the resuscitation team are advised to follow the CO_2_ trace and not absolute CO_2_ values.

CO_2_ detectors are routinely used to aid in the assessment of correct ET tube placement (9). The use of CO_2_ detectors reduces the time to confirmation of ET tube placement and has been endorsed in resuscitation guidelines (9, 64). Their use may be limited by false negative readings, during cardiopulmonary arrest and severe airway obstruction (65, 66). Employing quantitative capnography following ET tube placement also results in quicker and more accurate confirmation of correct placement when compared with clinical assessments (67, 68). There are no clinical trials comparing CO_2_ detector and capnography use to confirm correct ET tube placement.

The use of CO_2_ detectors during facemask ventilation has been shown to help determine airway patency on an almost breath-to-breath basis and can aid resuscitation teams in recognizing airway obstruction and leak during DR positive pressure ventilation (69–72). CO_2_ detectors can also verify the efficacy of positive pressure facemask ventilation during sustained inflations, as practiced in some centers (73). However, CO_2_ monitoring is not routine during mask ventilation. van Os and colleagues displayed the benefits of CO_2_ detectors in helping resuscitation teams to recognize airway obstruction in 24 very low birth weight infants during positive pressure support in the DR (69). Quantitative capnography during mask ventilation has been shown to improve CO_2_ elimination with the onset of an infant’s respiratory efforts; however, other authors have not found that it reduces the occurrence of hypocapnia or hypercapnia (74, 75). In a recent mannequin study, quantitative capnography was superior to CO_2_ detectors in improving efficacy of facemask ventilation (76). The results of the first clinical trial comparing the two methods of CO_2_ detection have yet to be published (CAPNO trial, unpublished, ISRCTN registration number: 10934870).

In the DR, the user controls ventilation pressures delivered to the infants’ lungs. The lungs of the preterm infant are susceptible to injury if exposed to high airway pressures. Immature animal models have shown that lung injury can occur after a few manual inflations at high pressure (77). On the other hand, facemask ventilation can be inadequate secondary to leak, even if the user is highly experienced (78, 79). In NICUs, ventilation adequacy can be assessed by *respiratory function monitors* (*RFMs*), which are incorporated into modern ventilators (80). They provide information not only on airway pressures but also on delivered tidal volumes. The monitor displays breathing pattern, tidal volumes, flow and pressure waves, and percentages of gas leak. RFMs have also been used to guide positive pressure ventilation in newborn resuscitations (81, 82). Schmolzer and colleagues found that RFM use during mask ventilation of preterm infants’ results in significantly less leak, more mask adjustments and a lower rate of excessive tidal volume given (82). RFMs have also been used in a RCT, which displayed improved ventilation with masks compared with nasal tubes during stabilization of preterm infants (83). However, the use and interpretation of a RFM can be technically challenging for many inexperienced users. Milner and colleagues recently surveyed 51 neonatal trainees who had used RFMs during preterm stabilization (84). They found that the usefulness of respiratory function monitoring was dependent on the trainee’s level of experience, and that appropriate responses to the RFM data were more frequent in the hands of senior clinicians compared with their junior colleagues. The composition of resuscitation teams may also play a role in the effectiveness of RFM use. Having an extra member present to interpret the RFM data and to advise on mask ventilation adjustments may improve its effectiveness but are difficult to justify in many centers with limited staff resources.

We look forward to further evidence on RFM use in preterm infants during DR stabilization, as they have the potential to monitor efficacy of ventilation while helping to protect premature lungs from unintended pressure related injury. However, at present, user difficulty remains a major limitation of RFM. In the meantime, we recommend that when positive pressure ventilation is initiated in the DR, consideration should be given to monitor ventilation efficacy. The superiority of which device has yet to be elucidated. Confirmation of ET tube placement should be confirmed with a CO_2_ detector as per current international guidelines.

## Neuromonitoring

As survival rates and short-term outcomes continue to improve for preterm infants, focus has shifted on neuroprotection strategies. The recent SafeBoosC trial suggests that brain oxygenation monitoring in the NICU results in a reduction in the percentage of cerebral hypoxia sustained by preterm infants (85). At present, assessment of neurological well-being in the DR is based on clinical assessment alone. Assessments of muscle tone and reflex irritability are incorporated into the Apgar score (3). The brain is the most vulnerable organ in newborn infants. As survival of the most immature infants increase, concerns have been raised about increased risks of adverse neurodevelopmental outcomes (86, 87). Resuscitative measures should aim for the best possible neurological outcomes and a non-invasive, continuous measurement of cerebral oxygenation and activity would be ideal, but these currently are not routine and their role has yet to be evaluated.

Studies that sought to introduce neurological monitoring into the DR initially focused on *cerebral blood flow* using Doppler measurements of cerebral or carotid arteries (44, 88–92). Monitoring was found to be technically difficult and did not provide continuous data (93). Furthermore, there is conflicting evidence on the role of cerebral Doppler in identifying impaired cerebral autoregulation and resultant abnormal cranial ultrasound findings (94, 95).

More recently, researchers have concentrated on *NIRS*, which provides non-invasive monitoring of cerebral tissue oxygenation (StO_2_) (96–109). NIRS utilizes the transparency of biological tissue to light in the near-infrared spectrum to measure tissue oxygenation (110). Cerebral tissue oxygen saturations in preterm infants have been shown to correlate well with superior vena cava flow and left ventricular output in the first days of life (111, 112). A number of studies have displayed the feasibility of obtaining cerebral oxygenation values using NIRS in the DR (93), and normative values for infants not requiring resuscitation have been published recently (103). However, the vast majority of these infants were full term infants. Table 2 summarizes the studies that have assessed NIRs in preterm infants in the DR.

**Table 2:** **Summary of preterm DR NIRS studies**.

Reference	Neonates	Number (*N*)	Design	Resuscitation group included	Observation
Fuchs et al. (109)	Preterm VLBW (<1500 g)	24	Observational	Yes All infants had SLI followed by CPAP	Increases in cerebral StO_2_ and HR preceded increases in SpO_2_ following SLI
Fuchs et al. (108)	Preterm VLBW (<1500 g)	51	Observational	Yes	Increases in cerebral StO_2_ values from 1 to 7 min of life before steady-state values reached Percentile charts produced
Binder et al. (100)	Late preterm 30 + 0 to 36 + 6 weeks	42	Observational	Yes	StO_2_ values were consistently higher in normal transitional group compared with stabilization group
Pichler et al. (103)	Term and preterm	*N* = 381 Preterm *n* = 27	Observational	No	Preterm infants post cesarean delivery had higher StO_2_ than term infants Percentile charts produced
Kenosi et al. (114)	Preterm <32 weeks	47	Observational	Yes	Infants requiring FiO_2_ > 3.0 had increased cerebral hypoxia, but no increase in cerebral hyperoxia compared to infants requiring FiO_2_ < 3.0
Pichler et al. (115)	Preterm <34 weeks	60	RCT	Yes	Reduction in cerebral hypoxia burden in group with NIRS and SpO_2_ monitoring in the DR

Cerebral NIRS in the DR remains limited to research studies, but emerging data suggest that it may have a significant role in preterm stabilization in the future (113). NIRS measurements are readily obtained and, in a recent study conducted by this group, the NIRS values were obtained within seconds of application of the device in the DR, in contrast to the variable time for pulse oximetry readings. Binder et al. performed NIRS on 49 preterm infants in the immediate newborn period (100). They reported different StO_2_ transition time courses for infants requiring respiratory support and those with normal transitions. Infants requiring respiratory support had lower StO_2_ values over the first 10 min of life before reaching similar steady-state levels as their counterparts. Fuchs et al. reported StO_2_ values for 51 infants weighing <1500 g (108). Low median StO_2_ values (37%) were reported at 1 min of life, which continuously rose to steady-state levels (61–84%) at 7 min of age. StO_2_ values did not differ in relation to the degree of resuscitation required in the DR, but it was noted that two infants with subsequent IVHs had StO_2_ values that were <10th percentile for their cohort. Kenosi et al. evaluated transitional cerebral NIRS values in preterm infants <32 weeks and found that preterm infants requiring >30% oxygen to maintain peripheral saturations had a significantly higher degree of cerebral hypoxia (114). All infants initially received a FiO_2_ of 0.3 and oxygen was titrated according to standard resuscitation guidelines. There were no differences in cerebral hyperoxia between the two groups. These findings suggest that some preterm infants may require a more rapid increase in oxygen titration in the DR. At present, NIRS remains in the realm of DR research, but as more studies emerge, we believe that it will have a future role in monitoring preterm infants and guiding oxygen titration during DR resuscitations. Pichler and colleagues have recently performed a pilot RCT, in which infants <34 weeks were randomized in the DR either to cerebral NIRS and SpO_2_ monitoring or SpO_2_ monitoring alone to guide titration of oxygen therapy (115). They found that additional NIRS monitoring significantly reduced the time that infants’ cerebral StO_2_ was <10th percentile in the first 15 min of life. There was no difference in rates of IVH or abnormal neurological assessments at discharge. Further trials are required to ascertain how oxygen therapy should be guided when StO_2_ and SpO_2_ values are both available in the DR.

Cerebral NIRS monitoring may also have a future in providing outcome measures for DR studies. A recent RCT randomized infants (28–33 + 6 weeks gestation) to receive either one to three sustained lung inflations (SLIs) (30 cmH_2_O for 15 s) followed by standard respiratory care, or standard respiratory care only (116). Cerebral tissue oxygenation values were similar for both groups over the first 15 min of life. However, cerebral blood volume (CBV) patterns differed between groups. CBV decreased in the control group over time, but remained static in the intervention group who received SLIs. The authors hypothesized that differences may have been caused by impaired venous return secondary to increased thoracic pressures during SLI, with resultant cerebral venous stasis. These findings highlight the importance of assessing cerebral hemodynamics during interventional DR studies.

*Electroencephalography* provides a real-time measure of electrocortical brain activity and has become more common in NICUs over the past two decades. In contrast to cerebral blood flow and NIRS, EEG has well-documented applications for the clinical management of newborn infants (117). Its usefulness includes monitoring infants with perinatal asphyxia (118–121), the detection of seizures (122–124), and more recently in assessing the long-term prognosis of both full term and preterm infants (125).

Despite its importance in monitoring the newborn brain in the NICU, only one study has assessed the feasibility of EEG monitoring in the DR. At present, we resuscitate infants at gestational ages that are at the borderline of viability without any information about brain activity. Pichler et al. performed aEEG (a modified form of EEG recording that filters, rectifies, amplifies, and compresses one to two channels of EEG activity) in infants >34 weeks gestation following elective cesarean section and achieved recordings after 3 min in some infants. However, continuous reliable data were difficult to obtain within their study of 63 infants (102). The two main factors restricting the introduction of EEG into the DR have been (1) the technical difficulties in acquiring reliable data in this setting and (2) the ability of people to interpret the data (93). Technical difficulties can be overcome especially given the recent introduction of easy to apply neonatal EEG sensors. The recent increase in research aiming to develop novel algorithms for neonatal EEG interpretation will also have an impact on DR brain monitoring in the near future (126). Further trials are required to assess the feasibility and utility of EEG recordings during transition for preterm infants.

## Video Recording

Video recordings provide objective measures of DR performance in the stabilization of preterm infants and can be utilized for education, research, and audits. Fifteen years ago, Carbine and colleagues reported that video recordings in the DR are feasible quality assurance tools (127). Video recordings can help assess neonatal teamwork in the DR, facilitate debriefings, and improve performance (128, 129).

Video analysis of newborn resuscitation and stabilization has shown that the availability of real-time information is less than optimal when compared with recommended guidelines (127, 130). In one study, <50% of high-risk infants had a pulse oximeter reading 60 s after transfer to the resuscitation table (130). These findings are important, as they emphasize the need to maintain clinical skills given that the availability of real-time physiological data from monitors can be delayed. In addition, they suggest that the feasibility of enacting current resuscitation guidelines should be constantly reassessed. Real-time video communication has been shown to improve adherence to neonatal resuscitation guidelines and may provide support for resuscitations in peripheral maternity centers (131). Nadler and colleagues assessed 19 neonatal newborn resuscitations, before and after the introduction of video recordings to facilitate debriefings. They found that all measures of teamwork improved after the intervention (129). Prior to introducing video recordings into DRs, ethical, legal, and practical obstacles must be overcome at a local level. These obstacles leave video recordings in the realms of research at present, despite their known practical benefits (132).

## Conclusion

Preterm infant monitoring during DR stabilization remains relatively basic when compared to the enhanced monitoring available in the NICU. At present, clinical assessments are the dominant paradigm; however, these have many limitations and so additional monitoring is required. All preterm infant stabilizations should have pulse oximetry monitoring to monitor HR and SpO_2_. ECG monitoring should also be available as an adjunct to ongoing clinical assessment and pulse oximetry when advanced resuscitation is anticipated. We believe that such monitoring should be initiated immediately after delivery. We also advise, while awaiting further research, that if positive pressure ventilation is commenced, either capnography or a RFM should be considered to monitor ventilation efficacy. Cerebral oxygenation monitoring, by NIRS and/or EEG, may have an important role to play in newborn transition. We look forward to further research in this area and recognize the challenges in performing such studies.

## Author Contributions

ED conceived and designed the review. DF drafted the initial manuscript. All authors (DF, CR, GB, and ED) critically revised the manuscript for important intellectual content, agreed on the final manuscript, and approved its submission for publication.

## Conflict of Interest Statement

The authors declare that the research was conducted in the absence of any commercial or financial relationships that could be construed as a potential conflict of interest.
